# Chronic heavy metal exposure in Yunnan Province disrupts intestinal defenses and increases susceptibility to pathogens: an integrative review

**DOI:** 10.3389/fpubh.2026.1794373

**Published:** 2026-05-08

**Authors:** Xinyu Li, Xin Zhao, Yuhan Yang, Keshu Zhao, Huijie Yang, Jiaxin Zhao, Yiying Zhang, Mingsheng Tang, Dongjie Li, Wei Zou

**Affiliations:** 1 School of Public Health, Kunming Medical University, Kunming, Yunnan, China; 2 Infection Control Office, Xi'an Public Health Center (Xi'an Emergency Medical Center), Xi'an, Shaanxi, China; 3 The First Affiliated Hospital of Kunming Medical University, Kunming, Yunnan, China; 4 School of Basic Medical Sciences, Kunming Medical University, Kunming, Yunnan, China

**Keywords:** heavy metals, immunological response, intestinal barrier, opportunistic infections, Yunnan Province

## Abstract

Chronic exposure to low levels of multiple heavy metals has emerged as a major public health concern in regions with complex geochemical backgrounds such as Yunnan Province, China. This study aimed to systematically elucidate the relationship between chronic composite heavy metal exposure and increased susceptibility to intestinal infection. To achieve this, we conducted a multidisciplinary synthesis integrating environmental monitoring data with evidence from cellular, immunological, and microbiological studies. Our analysis demonstrates that long-term exposure to lead, cadmium, and arsenic is associated with intestinal barrier dysfunction, gut microbiota dysbiosis, and altered immune responses, which together contribute to enhanced susceptibility to opportunistic pathogens, particularly *Pseudomonas aeruginosa*. Based on this integrated evidence, we propose a coherent causal framework linking multi-metal exposure to host vulnerability. Furthermore, this study provides strategic recommendations, including biological monitoring, environmental and nutritional interventions, and clinical management approaches, and highlights future research directions such as multi-omics investigations and model development. Importantly, this study has proposed a comprehensive interdisciplinary causal framework that links environmental exposure to the host-pathogen interactions within the specific regional geochemical background.

## Chronic combined heavy-metal exposure in Yunnan Province

### Unique geochemical background and anthropogenic drivers in Yunnan

Heavy-metal contamination in Yunnan arises from two major sources: (i) naturally elevated geological background levels and (ii) anthropogenic inputs from both unintentional and deliberate activities. Yunnan's baseline soil metal concentrations are relatively high, and agricultural practices and other human activities further elevate these levels (1). Together, Yunnan's geology and economic structure create persistent, population-level exposure to multiple metals. Across China, mining and smelting of non-ferrous metals are among the dominant contributors to heavy-metal pollution (2). In Yunnan, intensive Pb/Zn ore mining and smelting lead to enrichment of toxic trace elements—most notably arsenic (As), cadmium (Cd), and lead (Pb)—in soils and sediments (e.g., river sediments) (2). These activities release large quantities of metals into the environment, contaminating soils and dispersing via water flow and atmospheric transport, ultimately entering the food chain.

### Quantifying heavy metal pollution reports: lead (Pb), cadmium (Cd), and arsenic (As)

Quantifying human exposure requires measuring metal concentrations in environmental media (e.g., soil, water, and crops). For example, in Lanping Mining Valley—one of China's largest Pb/Zn mining areas—reported soil Pb concentrations are approximately 56-fold higher than the global average (3). In a survey across 11 cities in Yunnan, mean soil Cd and Pb were reported as 1.31 mg/kg and 64.17 mg/kg, respectively, exceeding local background levels. In highly contaminated locations, average Pb concentrations reached ~40 mg/kg in Nujiang Prefecture, and mean Cd reached ~4.03 mg/kg, with Cd exceedance in Qujing reported as high as 92.31% (4). Soil metals can be taken up by crops and accumulate in edible tissues, forming a key dietary exposure pathway. In the same 11-city survey, mean Pb and Cd in edible crop parts were 0.20 mg/kg and 0.08 mg/kg, respectively. In heavily polluted areas near the Gejiu tin mine, Pb and Cd levels in crops reportedly exceeded national food-safety limits by 12.10-fold and 16.16-fold, respectively (4). Because leafy vegetables and legumes tend to accumulate Pb and Cd more readily, populations relying heavily on these foods may face higher dietary exposure. Overall, heavy-metal pollution in Yunnan shows strong geographic heterogeneity and involves complex mixtures that may pose ecological and human-health risks.

### Population exposure routes and health-risk assessment

In Yunnan, the main exposure routes for residents include consumption of crops grown on contaminated soils and intake of potentially contaminated surface or groundwater. Importantly, this exposure pattern is typically chronic, low-dose, and mixture-based, rather than acute, high-dose exposure. Such long-term exposure may impose sustained physiological stress, with health effects that can be subtle and cumulative over time (2). Children are often considered a particularly vulnerable subgroup due to developmental susceptibility, higher absorption rates, and behavior-related exposure patterns. More direct human exposure evidence from Yunnan is also available. Cd measurements in blood, diet, and drinking water among Pumi residents in Lanping, showing that dietary and waterborne exposure contributed to internal Cd burden in a mining-affected setting (5). In another study from the Huodehong Pb/Zn mining area, residents living near the mine had higher Cd and Pb levels in scalp hair than those in a comparison area, and dietary intake from locally grown crops was identified as a major exposure pathway with substantial non-carcinogenic and carcinogenic risk (6). Long-term environmental surveillance data, although still limited, further support continued regional concern; a 20-year study of the Manwan Reservoir in the Lancang River documented persistent temporal and spatial variation in multiple heavy metals in surface water, indicating that metal contamination in Yunnan should not be viewed only as a short-term or isolated event (7).

### Regional epidemiological relevance and current limitations

To better justify the regional focus of this review, it is important to distinguish among environmental contamination, exposure plausibility, human biomonitoring, and direct population-level health evidence. In Yunnan, the currently available literature extends beyond soil-crop contamination alone. Biomonitoring studies from mining-affected areas have detected elevated internal burdens of Cd and Pb in local residents (5), while a community survey of 1,379 children and adolescents from a mining-affected area of Yunnan reported a mean blood Pb level of 82.87 ± 66.97 μg/L and a Pb poisoning rate of 24.7%, with higher risk among boys, preschool-age children, and those living closer to mining areas (8). In addition, a large study of five Cd-polluted areas in China found a positive association between urinary Cd and renal tubular dysfunction, supporting the plausibility of measurable health damage in chronically exposed Chinese communities, although that study was not designed specifically around intestinal infection outcomes (9). These findings still do not establish a direct causal relationship between heavy-metal exposure in Yunnan and increased susceptibility to opportunistic intestinal infection. They do, however, strengthen the epidemiological basis for treating Yunnan as a geographically relevant case study in which environmental contamination, human biomonitoring, and observed adverse health effects collectively justify concern regarding downstream intestinal and immune consequences.

### Methodology of literature retrieval and study selection

This narrative review was based on a structured literature search and study selection process. Relevant studies were retrieved from PubMed, Web of Science, and Scopus and supplemented by manual screening of the reference lists of related articles. The literature search covered studies published from 1998 to 2025. The search strategy used combinations of keywords related to heavy metal exposure and intestinal host defense, including “heavy metals,” “lead,” “cadmium,” “arsenic,” “intestinal barrier,” “tight junction,” “gut microbiota,” “dysbiosis,” “immune imbalance,” “opportunistic pathogen,” and “*Pseudomonas aeruginosa*,” combined with Boolean operators such as “AND” and “OR.”

**Figure 1:**
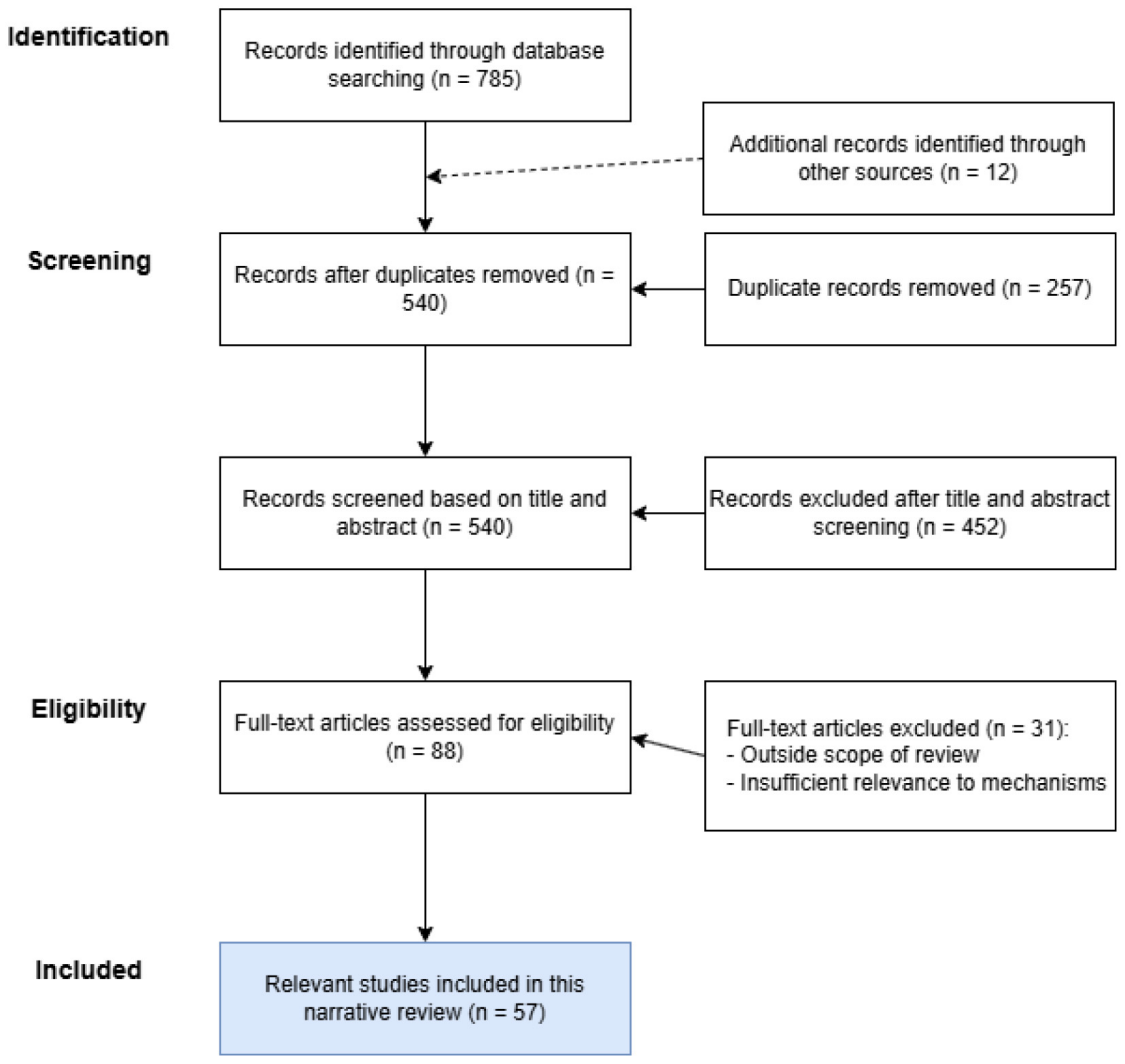
Flow diagram of the literature search and study selection process.

The review focused on evidence concerning chronic exposure to heavy metals, particularly Pb, Cd, and As, and their relationships with intestinal barrier dysfunction, gut microbiota dysbiosis, immune imbalance, and susceptibility to opportunistic pathogens. The inclusion criteria were as follows: (1) original research articles or relevant review articles published in peer-reviewed journals; (2) studies addressing at least one of the following topics: heavy metal exposure, intestinal epithelial barrier injury, gut microbiota alteration, immune dysregulation, or increased susceptibility to opportunistic infection; (3) studies involving human populations, animal models, cell models, or mechanistic experimental systems relevant to the topic of this review; and (4) articles providing information directly relevant to the mechanistic framework discussed in this manuscript. The exclusion criteria were as follows: (1) duplicate publications; (2) conference abstracts, editorials, commentaries, letters, or publications without sufficient primary or review content; (3) studies not relevant to the scope of this review; (4) articles with insufficient relevance to the proposed mechanisms linking heavy metal exposure to intestinal defense disruption and pathogen susceptibility; and (5) studies for which the full text was unavailable or did not provide adequate information for qualitative synthesis. After duplicate removal, records were screened based on titles and abstracts, and potentially relevant studies were subsequently assessed through full-text review for eligibility. The overall study selection process is summarized in Figure 1.

## Heavy metal-induced intestinal barrier dysfunction

### The destruction of tight junctions by heavy metals

Multiple layers of the intestinal barriers form the overall structure of a functioning gut. The structure from outside to inside is as follows: mucus layer, single layer of intestinal epithelial cells (IECs), and the apical junctional complex located at the top of the cells. This complex contains tight junctions (TJs), adherens junctions, and desmosomes. Of the three components of this aggregate, TJs have been studied most extensively and serve as the functional barrier, controlling and determining the passage of material into and out of the intestinal lumen by regulating permeability. TJs form a zipper whose teeth hold the adjacent cells together, thereby becoming the limiting factor in the electrical and chemical regulation of IEC function (10). Intestinal barrier dysfunction is a multifactorial pathological condition characterized by structural and functional impairment of the intestinal barrier. Increased intestinal permeability, often described as “leaky gut,” represents one of its key functional manifestations (11). Intestinal barrier dysfunction is primarily caused by disruption of tight junctions, which can be directly induced by heavy metals (12). Similarly, As decreases Claudin-1 and Claudin-5 expression, reducing transepithelial electrical resistance (TEER) in HT-29 cells (13). Pb exposure also lowers ZO-1 and occludin levels in colonic tissue, causing dysbiosis, inflammation, and tissue damage (14). Yunnan's residents are highly likely to be exposed to multiple metal exposures; thus, the interactions between mixtures of metals are critical to understanding potential health risks. There are few studies directly addressing the toxicology of the indicators of combined Pb, Cd, and As mixtures; however, evidence does suggest that these three metals have a synergistic effect that damages health. For example, studying Cd and ochratoxin A co-exposure in Caco-2 cells resulted in decreased Claudin-1, Occludin, and E-cadherin expression and a synergistic effect (15). Additionally, combined Cd and As exposure has also been shown to cause synergistic reproductive and developmental toxicities in *Caenorhabditis elegans* (16). Heavy metals create not only cell necrosis due to low concentrations but also a specific targeting of the principal molecular components responsible for maintaining barrier function. While Cd coordinates the mislocalization of ZO-1 and occludin (17).

### Impact of heavy metal exposure on human intestinal health

There has been a substantiating body of epidemiologic research linking chronic exposure to heavy metals with impaired gastrointestinal integrity among various human populations. Large-scale biomarker monitoring studies have demonstrated that higher concentrations of As, Cd, and Pb in internal tissues correlate significantly with gastrointestinal disturbances and pro-inflammatory biomarkers of both systemic and intestinal inflammation. For example, recent analyses derived from the National Health and Nutrition Examination Survey (NHANES) have shown significant positive correlations between elevated urinary concentrations of Cd and As and pro-inflammatory biomarkers, as well as increased intestinal permeability at the population level, indicating disruption of the integrity of the gastrointestinal barrier (18, 19). Concurrently, the emerging field of human microbiome research has also demonstrated the role of the gut as a key organ for heavy metal/environmental exposure. In cross-sectional and cohort-based studies, heavy metal exposure has been associated with decreased gut microbial diversity and compositional changes in the gut microbiota, including loss of beneficial commensal organisms and an increase of potentially pathogenic microbial taxa (20, 21). Dysbiosis, as identified in these studies, has been associated with altered metabolic profiles and decreased ability to establish colonization resistance, putting individuals at risk for opportunistic infections.

Additionally, recent epidemiologic studies highlight the need to consider the impacts of mixed-metal exposures as they are more representative of real-world environmental exposures. One study has indicated that co-exposure to multiple heavy metals produces distinct microbiome profiles when compared to single metal exposure (22). Another recent study demonstrated enhanced oxidative stress and inflammatory response in exposed populations exposed to Cd and Pb, suggesting that the effects of mixed metal exposures may be additive or synergistic (20). Cumulative effects on gastrointestinal health were observed in numerous studies of human populations residing in mining and industrial areas throughout China and Southeast Asia that had low-dose, long-term exposure to mixed metals (23). These investigations provide compelling support for the need to assess potential exposure scenarios for heavy metals as complex mixtures, rather than solely as individual contaminants.

## The intestinal epithelial barrier and its molecular basis for damage

### Induction of oxidative stress and activation of pro-inflammatory signals

Reactive oxygen species (ROS) are a major source of cellular oxidative stress to cells exposed to heavy metals. The presence of high concentrations of ROS may disrupt the normal redox balance of a cell and deplete the amount of antioxidant defenses available in that cell [e.g., glutathione (GSH)] (24). Heavy metals induce oxidative stress that mediates damage to tight junctions. One example is seen in HT-29, a human colon cancer cell line, where the antioxidant, N-acetylcysteine (NAC), was able to ameliorate both the downregulation of the expression of claudins (tight junction proteins) and the accompanying reduction in TEER (13). Oxidative stress is controlled, in part, through the KEAP1-NRF2 regulatory pathway and is considered part of the overall cellular mechanism with which oxidative stress is managed (25). NRF2 is able to be activated by heavy metals and, once activated, is translocated to the nucleus where it induces the transcription of many genes that will attempt to prevent cellular damage due to ROS. The chronic exposure of heavy metals may lead to long-term activation of the KEAP1-NRF2 pathway. Eventually, following chronic activation, NRF2 may lose its ability to function or become dysregulated. Among other findings, transcriptomic analyses of low and non-lethal Cd exposure have also demonstrated significant changes in gene expression relating to oxidative stress [Nrf2 and metallothioneins (MT)] (26). Additional studies have shown that heavy metals cause an increase in mitochondrial dysfunction and open the mitochondrial permeability transition pore (MPTP) in response to heavy metal-induced cellular injury, which may lead to apoptosis and, subsequently, sloughing off of epithelial cells from the intestinal barrier (27). The sloughing and shedding of epithelial cells from the intestinal barrier create gaps in the intestinal barrier, allowing for increased permeability between the intestinal lumen and systemic circulation. The NF-κB pathway is essential for regulating inflammatory immune responses. Inflammation is caused by the activation of the NF-κB pathway by heavy metals and may result in the production of numerous inflammatory cytokines and chemokines, which aggravate the inflammation and damage of the intestinal mucosa (28). Furthermore, oxidative stress caused by heavy metal exposure can lead to the activation of intracellular pathways responsible for producing proinflammatory signals. For example, the downregulation of Claudin proteins induced by arsine has been shown to be mediated through the phosphorylation of both p38 MAPK and NF-κB pathways (13). Heavy metals may also cause the phosphorylation of p38 MAPK in Pb-treated neural progenitor cells (29).

### The mechanism differences of different heavy metals

Numerous studies support that oxidative stress, mitochondrial dysfunction and inflammatory signaling are elements that contribute to impaired intestinal barrier function caused by heavy metals, however the existing literature tends to consider these pathways separately and has not sufficiently quantified their relative contributions. The metals Pb, Cd and As display unique but overlapping molecular profiles. There is considerable literature indicating that Cd induces mitochondrial dysfunction, upsets calcium homeostasis and mislocates the tight junction proteins, ZO-1 and occludin, damaging epithelial integrity at concentrations below those known to be cytotoxic (17). In comparison, As differentially affects transcriptional regulation of tight junction components and stimulates redox-sensitive signaling pathways such as the MAPK and NF-κB signaling cascades (30). Finally, although Pb does not directly target these junctional complexes, it has been linked to dysbiosis of gut microbiota and disruption of epithelial barriers through inflammation secondary to dysbiosis (31). The limited number of studies comparing metal interactions in co-exposure scenarios suggest that there may be either an additive or synergistic effect due to complementary mechanisms of action on epithelial structure, mitochondrial function and immune regulation. For example, co-exposure models show enhanced disruption of tight junction proteins and a greater degree of oxidative and inflammatory responses when compared to single-metal exposures (15). Unfortunately, the previously stated types of studies have primarily been completed *in vitro* and in animal studies and additional work is needed to determine how well these results apply to humans exposed to mixtures found in the real world. One of the areas requiring additional research centers on the relationship between oxidative stress and inflammatory signaling. Specifically, even though most consider ROS to initiate these processes, there is a significant amount of literature showing concurrent activation of inflammatory pathways, making it difficult to ascertain whether oxidative stress is the main cause of injury or simply an enhancer of injury to tissue (32). Therefore, these studies indicate the need for integrated, comparative frameworks to distinguish between metal-specific effects vs. overall effects of mixtures, to determine temporal relationships between oxidative stress and inflammatory signaling and, to utilize physiologically relevant levels of exposure to provide more accurate assessments of risk to human health.

The damage inflicted by heavy metals on intestinal epithelial cells is not a one-way stepwise linear process. Instead, it is a cyclical interplay of three major pathological pathways (oxidative stress, mitochondrial dysfunction, and activation of pro-inflammatory signaling), interacting with one another and reinforcing each other in a manner that contributes to a chronic state of cellular damage from chronic and low-level exposure. Mitochondrial dysfunction results from heavy metal exposure, where dysfunctional mitochondria create a greater amount of cellular ROS. Elevated levels of ROS activate NF-κB and pro-inflammatory pathways. The inflammation created by NF-κB causes further damage to mitochondria. Table 1 depicts some of the ways in which various heavy metals (Pb, Cd, As) cause damage to the intestinal epithelium through their impact on tight junctions of intestinal epithelium, oxidative stress, damage to mitochondria, and damage to signaling pathways.

**Table 1:** The molecular mechanism of intestinal epithelial damage induced by heavy metals.

Cell processes/components	Specific target/indicator	Pb	Cd	As	References
Tight junctions	Claudin-1, Claudin-5	-	-	Reduce	(13)
	Occludin, ZO-1	Reduce	Reduce and locate anomalies	-	(14, 17)
	TEER	-	Reduce	Reduce	(13, 17)
Oxidative stress	ROS	Produce	Produce	Produce	(12, 14)
Mitochondria	ATP	Restrain	Restrain	Restrain	(12, 27)
	MPTP	Open and trigger apoptosis	Open and trigger apoptosis	Open and trigger apoptosis	(27)
Signal channel	NF-κB	Activate	Activate	Activation and nuclear translocation	(13, 28)
	p38 MAPK	Activate	Activate	Phosphorylation activation	(13, 29, 30)
	KEAP1-NRF2	Activate	Activate	Activate	(25)

TEER, transepithelial electrical resistance; ROS, Reactive oxygen species; GSH, glutathione; ATP, Adenosine Triphosphate; MPTP, mitochondrial permeability transition pore.

## Causes of dysbiosis within the intestinal microbial community

### Heavy metals as drivers of gut microbiota dysbiosis

Long-term exposure to heavy metals such as Pb, Cd, and As significantly disrupts the composition, structure, and diversity of the gut microbiota, thereby driving dysbiosis (21). In studies using animals, it has been shown that subchronic exposure of mice to Cd results in a marked decrease in abundance of Firmicutes in both feces and cecal contents and alters the overall microbial structure (33). Similarly, exposure of mice to Pb results in the alteration of gut microbiota with an increase in the Firmicutes/Bacteroidetes (F/B) ratio correlated with metabolic aberrations (34). There is a multi-faceted mechanistic pathway for how heavy metals influence gut bacterial composition and diversity through direct toxic actions and indirectly altering the intestinal microenvironment. At the direct toxicity level, heavy metals produce ROS that damage bacterial DNA, proteins, and lipid membrane and therefore inhibit the growth of certain microbial populations (21). At the same time, heavy metals activate stress response (SOS) pathways in microorganisms that alter the expression of virulence genes and consequently alter metabolic activity (35). At the indirect level, co-exposure to multiple metals significantly reduces body weight gain in male mice and leads to gut microbiota changes, including decreases in *Lactobacillaceae* and *Lachnospiraceae* and an increase in *Desulfovibrionaceae* (36). The gut microbial composition changes from heavy metal exposure are not limited to changes due to compositional abundance, they also affect many important key metabolic pathways of gut microorganisms such as energy production and detoxification processes (21).

### Dysbiosis exacerbates barrier damage and metal absorption

Maintaining gut barrier integrity requires the presence of healthy gut microbiota. The gut microbes, and the metabolites produced by the gut microbes [in particular short-chain fatty acids (SCFA)], provide the energy source required by the intestinal epithelial cells, regulate the expression of tight junction proteins, and contribute to the maintenance of intestinal immune homeostasis (35). Butyrate, one of the SCFA produced by gut microbiota, increases the expression of tight junction proteins (ZO-1, Occludin, Claudin) through the activation of G-protein coupled receptors and inhibition of histone deacetylases, resulting in the enhancement of intestinal barrier function (35). A study found that after the gut microbiota had been disrupted in mice treated with antibiotics, the accumulation of both elements was significantly higher (30.9–119% higher for cadmium, 30–100% higher for arsenic in the liver) among mice fed cadmium- and arsenic-contaminated rice compared with those with a healthy gut microbiota. This increase in absorption of cadmium and arsenic is closely associated with the changes in mucus layers and the depletion of tight junction proteins associated with dysbiosis and increased intestinal permeability (37). Thus, dysbiosis caused by exposure to heavy metals is not only a secondary effect, but also plays a key role in worsening gut permeability and, therefore, absorption of heavy metals from the gut. Gut microbes limit heavy metal toxicity by several potential mechanisms, including: biosorption and chelation (immobilizing metal ions within the intestinal tract) through the production of microbial metabolites, biotransformation of heavy metals (transforming heavy metals into different valence states to reduce their toxicity), and immunoregulation by producing various microbial metabolites (e.g., SCFA, tryptophan derivatives). These metabolites promote T helper 17 (Th17) and regulatory T (Treg) cell differentiation and immune homeostasis and maintain the integrity of the intestinal barrier (i.e., heavy metal exposure reduces these metabolites causing a Th17/Treg imbalance in Treg differentiation gene expression) (35). Other studies indicate that germ-free mice accumulate significantly greater amounts of heavy metals in blood and other target organs (either liver or kidney) after ingestion of drinking water containing Cd or Pb when compared to specific-pathogen-free (SPF) mice with normal microbiota (38). These results indicate that the healthy commensal microbiota present within the intestinal tract protects the host from entry of heavy metals.

Alterations in gut microbiota composition may serve as a more sensitive biomarker of heavy metal toxicity that appears earlier than clinical symptoms. For example, among residents long-term exposed to multiple metals in mining and smelting areas, the composition of the gut microbiota (*Lachnospiraceae, Eubacterium*, and *Bacteroides*) changed significantly (39). Furthermore, diet, as a key factor shaping the gut microbiota, plays an important role in modulating metal toxicity. A high-fat diet exacerbates liver and kidney damage in mice exposed to As, Cd, and Pb, likely because a high-fat diet reduces the ability of the gut microbiota to excrete heavy metals (40). This finding suggests that the dietary pattern of a population can alter susceptibility to environmental toxicants. Therefore, in addition to reducing direct pollutant intake, adjusting dietary structure to cultivate a microbiota that is more resistant to and buffers against metal toxicity may become an important but currently underestimated public health intervention strategy. Probiotic and prebiotic interventions show promise in alleviating heavy metal toxicity through mechanisms such as adsorption of metal ions, modulation of microbial composition, restoration of SCFA levels, and reduction of inflammatory responses (41). In summary, heavy metal exposure triggers dysbiosis through the multiple mechanisms described above, and this dysbiosis in turn exacerbates intestinal barrier dysfunction through metabolite depletion, impaired epithelial repair, immune imbalance, and amplification of inflammation. Table 2 identifies the various ways that heavy metals have an effect on the intestinal microbiota.

**Table 2:** The alterations in gut microbiota and local immune responses induced by heavy metals.

Category	Indicator	Main changes (Pb/Cd/As/Mixed metal exposure)	Functional consequences	References
Microbiota	Microbial diversity	Decreased	Reduced colonization resistance, disrupted microbial structure	(21, 33)
	*Firmicutes*	Significantly decreased abundance; increased F/B ratio	Energy metabolism disorder, associated with metabolic dysfunction	(33, 34)
	*Bacteroidetes*	Increased F/B ratio (relative change)	Microbial imbalance	(34)
	*Lactobacillaceae*	Decreased	Reduction of beneficial bacteria, impaired barrier protection	(36)
	*Lachnospiraceae*	Decreased	Reduced SCFAs production, impaired intestinal barrier	(36, 39)
	*Desulfovibrionaceae*	Increased	Increased pro-inflammatory bacteria, elevated risk of intestinal inflammation	(36)
Immune response	Th1/Th2 balance	Promote Th2 and inhibit Th1	The anti-infection ability dependent on Th1 is weakened.	(44, 45)
	Th17/Tregs balance	Shift toward Th17 (imbalance)	Breakdown of immune tolerance, exacerbated inflammation	(35, 49)
	AhR pathway	Suppressed (due to reduced microbial metabolites)	Impaired immune homeostasis	(44, 45)

## Mechanism of violation of host immune system balance

### Heavy metals and immune dysfunction

Gut-Associated Lymphoid Tissue (GALT) represents the largest and most complex immune organ in the body and operates to maintain a delicate balance between establishing immune tolerance to the various commensal microorganisms and food antigens while simultaneously providing an effective defense against invading pathogens (42). This balance between being tolerant of a large group of otherwise harmless microbes and recognizing pathogens is largely influenced by the functional activities of pro-inflammatory Th17 cells and anti-inflammatory T regulatory cells (Tregs). It is the cytokines IL-17A/F and IL-22 that Th17 cells make that protect against pathogens. These are very important for fighting bacterial and fungal infections and keeping the epithelial barrier working. The activities of Tregs, on the other hand, limit the development of autoimmune and inflammatory diseases through their ability to suppress excessive activation of the immune response (42). Heavy metals are very potent immunotoxins that can directly inhibit the normal function of the immune system (43). Exposure to Pb has been proven to cause a decline in cellular immune function, thereby transforming the immune response from a Th1-type dominated response to a Th2-type dominated response characterized by antibody production and atopy. Lead can interfere with the reactivity of T cells. It promotes the development of Th2 cells preferentially and inhibits the development of Th1 cells (44, 45). The result of this is a decrease in the host's ability to clear specific classes of pathogens. The combination of Pb, Cd, As, and mercury can also lead to substantial reductions in lymphocyte populations, as well as changes in the innate immune response profiles, creating a general impairment of the host's ability to fight pathogens (43). Table 2 lists heavy metal effects on the immune system.

### Barrier disruption and dysbiosis serve as triggers for pathological Th17 activation

**Figure 2:**
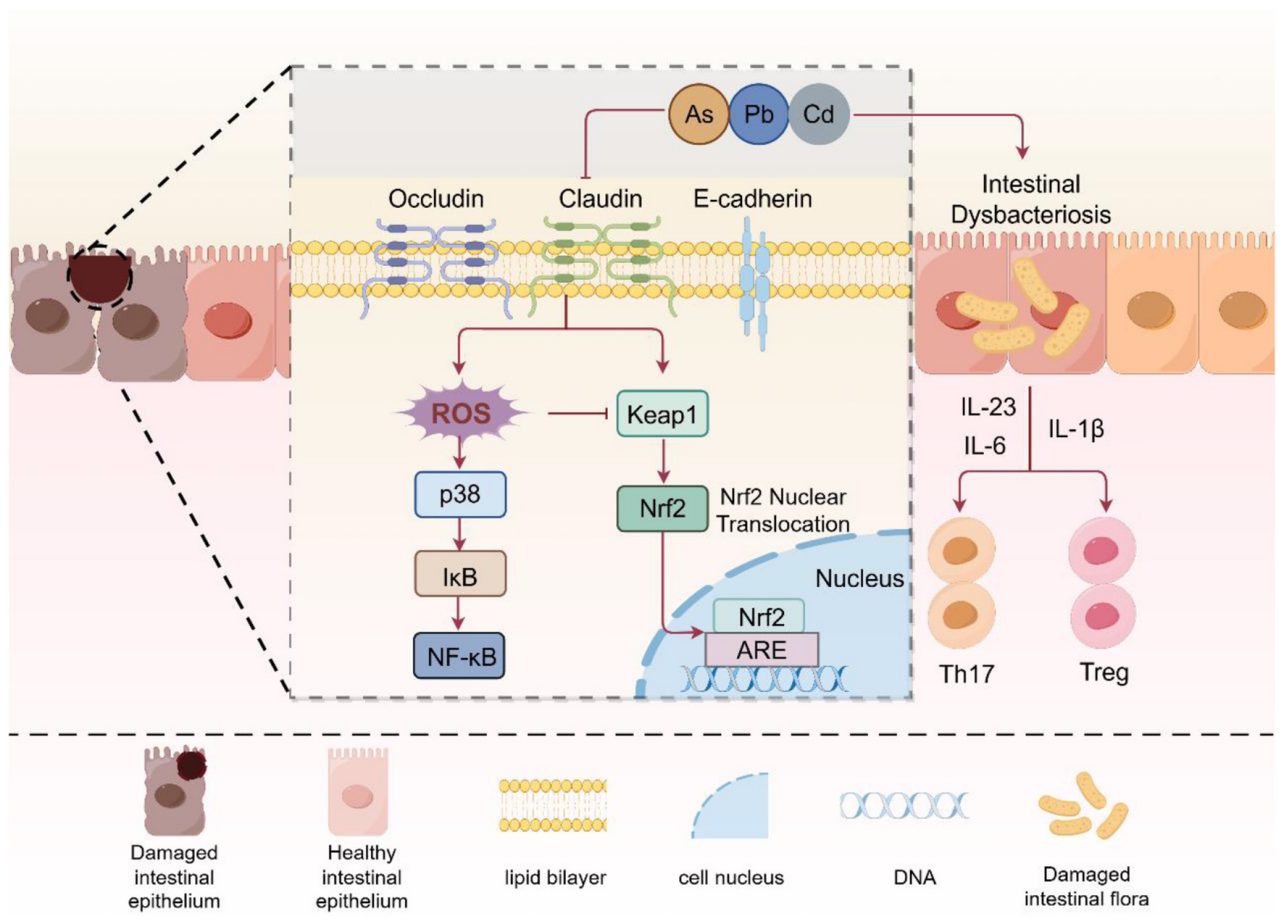
Mechanisms of intestinal barrier disruption and dysbiosis. Heavy metals inhibit the expression of key tight junction proteins Occludin, Claudin and E-cadherin in the intestine, promoting the imbalance of intestinal flora. Subsequently, through the p38, IκB, and NF-κB inflammatory pathways, they promote oxidative stress. Among them, the Keap1-Nrf2 pathway can defend against the damage caused by oxidative stress to cells. Specifically, under heavy metal exposure, Keap1 is activated, thereby promoting the translocation of NRF2 to the nucleus, binding to the antioxidant response element ARE, and inducing the expression of a series of antioxidant and detoxification genes to protect the cells. On the other hand, the destruction of intestinal flora leads to an increase in the secretion of IL-6, IL-23, and IL-1β, thereby promoting the transformation of Th17 and Treg cells to a pathological state (Create with Figdraw: https://www.figdraw.com/).

A primary function of the intestinal epithelial barrier is to maintain homeostasis between the host and microbial populations that enter through the epithelial layer of the gut, using various mechanisms such as the tight junction proteins that create an impermeable barrier, the mucosal layer, antimicrobial peptides present within the intestinal lumen, and the immune system functioning in both an innate and adaptive manner. In addition, gut microbiota supports immune tolerance while mitigating excessive inflammatory responses (46). Once epithelial barrier function has been compromised, microbiota-derived products such as lipopolysaccharide (LPS) or bacterial metabolites may enter the submucosal tissue through the epithelial barrier and activate innate immune cells and secrete pro-inflammatory cytokines, creating an altered immune microenvironment (47). At the same time, dysbiosis negatively impacts the ecological homeostasis of the gut, resulting in the loss of protective mechanisms that promote ecological homeostasis through the competitive inhibition of pathogens and the production of SCFA (48). The combination of these events produces a situation in which antigen presenting cells (e.g., dendritic cells) come into contact with an increased number of microbial antigens, leading to the secretion of cytokines such as IL-6, IL-23 and IL-1β that promote Th17 differentiation and drive Th17 cells into a pathological phenotype. In environments characterized by barrier disruption and dysbiosis, Th17 cells are characterized by an increased inflammatory potential (e.g., co-expression of IFN-γ and GM-CSF, loss of anti-inflammatory IL-10 effects, participation in chronic inflammation and autoimmunity, and increased susceptibility to infection from pathogens) (49). The pathogenesis of various chronic inflammatory diseases, such as inflammatory bowel disease (IBD), is closely related to this pathological Th17 (42). In addition, metabolites produced from pathogenic microorganisms or commensal bacteria may also help direct the fate of Th17 cells into a pathological phenotype (Figure 2).

The immune system, which has been directly weakened, is forced to respond to a self-threat created by intestinal barrier dysfunction, ultimately leading to functional dysregulation or failure. This chronic, low-grade activation state of the GALT, driven by barrier disruption and dysbiosis, may place the immune system in two dangerous situations: either being pre-activated and responding with a destructive and excessive inflammatory reaction when encountering a true pathogen, or prolonged overstimulation leading to immune cell functional exhaustion, rendering it unable to respond effectively to new threats. The final outcome, whether it be destructive excessive inflammation or immune paralysis, will severely impair the host's ability to eliminate opportunistic pathogens.

## The increased susceptibility toward azotobacteria sp., for instance, *Pseudomonas aeruginosa*

### How the changes induced by metals are beneficial to the pathogen

*Pseudomonas aeruginosa* is a gram-negative bacterium found in many natural environments. Pseudomonas does not cause serious disease in healthy persons; however, it is an extremely aggressive opportunistic pathogen and can produce serious or fatal infections in individuals with immune dysfunction or physical barrier degradation (50). *Pseudomonas aeruginosa* can produce many different exotoxins (ExoS, ExoT, and ExoU) and metabolic end products (elastase, phospholipase, and pyocyanin) that interfere with host cells (cells of the body), inhibit the immune system from responding, and allow for invasion of new tissue (51). Additionally, as part of its life cycle, *Pseudomonas aeruginosa* can form biofilms, which allows it to create a physical barrier from the immune system and significantly increases its resistance to antibiotics and the immune system (51). Heavy metals damage the intestinal epithelial barrier, creating a direct route for *Pseudomonas aeruginosa* to invade. As tight junctions fail, *Pseudomonas aeruginosa* is able to migrate through these destroyed tight junctions and into previously sterile submucosal tissue and obtain greater sources of nutrients and evade the surface-based mechanisms of clearance. The healthy microbiota contributes to a strong colonization resistance; this resistance is generated by competing for nutrients and space with pathogens as well as by creating antimicrobial compounds. When heavy metals suppress the beneficial bacteria that grow and thrive in the human gastrointestinal tract, the colonization resistance provided by these beneficial microbes is reduced, thus allowing the former commensals to create an environment for pathogenic *Pseudomonas aeruginosa* to flourish.

### 2 The immune system is unable to eliminate *Pseudomonas aeruginosa*

Some heavy metals may serve to directly increase the pathogenicity of *Pseudomonas aeruginosa*. For example, chronic exposure to zinc at elevated levels will broadly induce virulence factor production by *Pseudomonas aeruginosa* and increase the ability of *Pseudomonas aeruginosa* to transition from a commensal to pathogenic form as well as increase its ability to damage the mucosa and induce inflammation in murine models (52). While zinc is different than Pb, Cd, or As, this information shows that metal contamination could serve as a signal that directly acts upon pathogenic organisms. There is also evidence that heavy metal contamination in the environment functions as a selective pressure on bacteria and subsequently enables the emergence of bacterial strains that are not only highly virulent but also highly resistant to metals (53). Therefore, heavy metals present in the environment are a major public health concern, since they allow the co-selection of bacterial strains that are resistant to metals and antibiotics. This is possible since the genes responsible for both types of resistance are typically found on the same mobile genetic elements, including plasmids (54).

**Figure 3:**
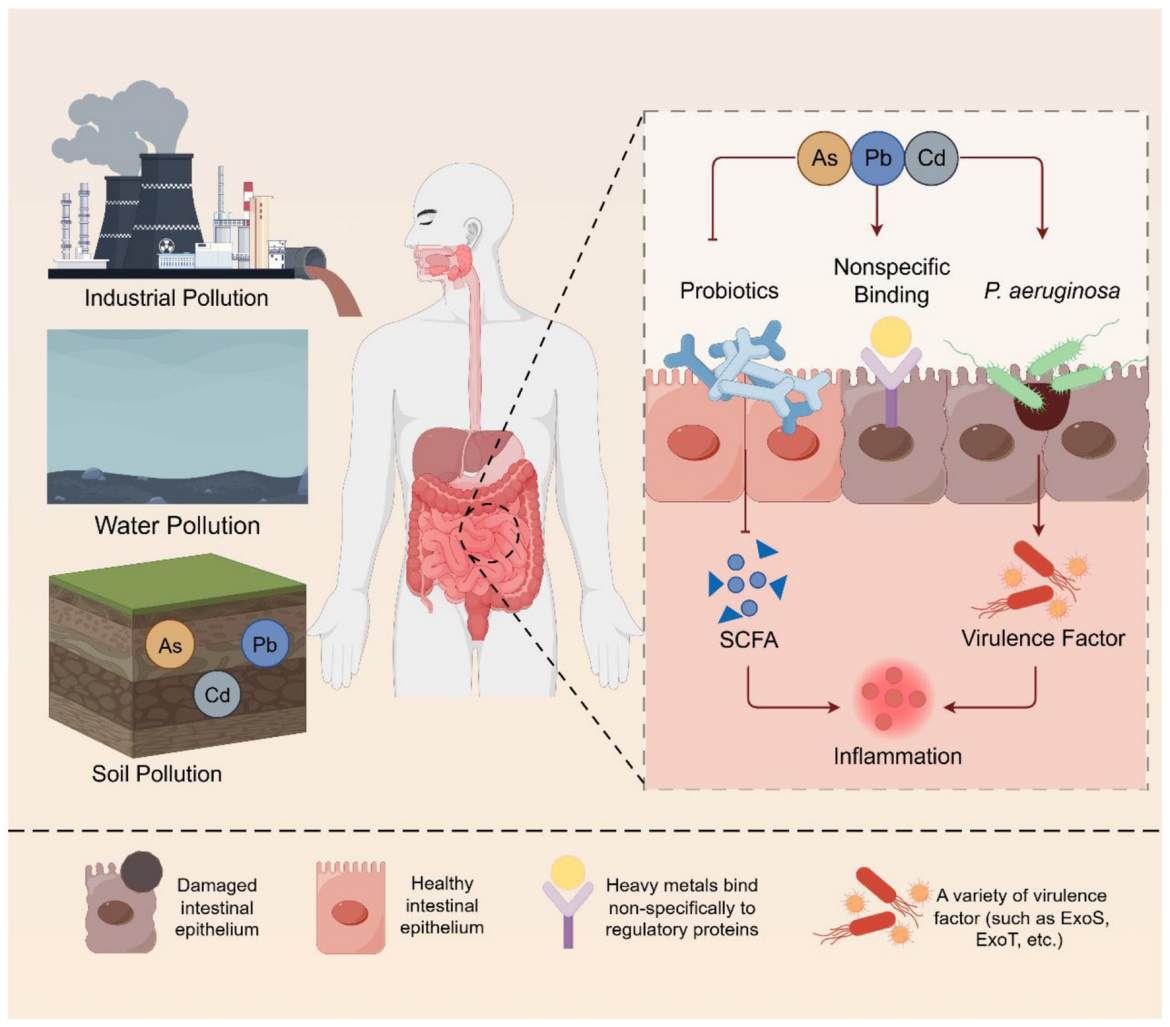
Complex heavy metals disrupt intestinal defense and increase susceptibility to pathogens. Industrial, water and soil pollution in the environment can release a large amount of heavy metals. Long-term exposure to such an environment by the human body can inhibit the growth of beneficial intestinal bacteria and suppress the vegetation of the intestinal flora. Heavy metals can non-specifically bind to regulatory proteins. *Pseudomonas aeruginosa* and other opportunistic pathogenic bacteria are more likely to invade the body, thereby inhibiting the growth of short-chain fatty acids and promoting the secretion of toxic factors. Eventually, it leads to intestinal inflammation in the human body (Create with Figdraw: https://www.figdraw.com/).

Once *Pseudomonas aeruginosa* has gained successful entry into the host, the host's ability to clear the infection is typically impaired. The combination of the immunotoxicity from the increased levels of heavy metals plus the impaired function of the GALT suggests that the host's immune system will not be capable of effectively clearing the invading pathogens (55). In addition, nutritional immunity dysfunction may have an important role in this process as well. Nutritional immunity is a method of innate defense deployed by the host that works by denying invading pathogens access to essential nutrients through regulated availability of essential trace metals to the body (56). To achieve this, the host produces multiple types of proteins (e.g., metallothionein, transferrin, and calprotectin) that chelate and sequester trace metals. However, when non-essential toxic heavy metals such as Pb, Cd, and As are present in high levels, they can significantly disrupt the finely tuned regulatory system that maintains metal homeostasis. These toxic heavy metals can bind sequelae to the regulatory proteins, causing saturations to their binding sites and inhibiting normal expression and activity (26). This results in the host no longer being able to adequately implement nutritional immune mechanisms, thus allowing *Pseudomonas aeruginosa* to receive an adequate supply of the crucial nutrients that support its growth and reproduction (Figure 3).

## Comprehensive analysis and recommendations

### Conceptual framework integrating established mechanisms and inferred links

The available literature supports several individual components of the proposed framework, although the full cascade has not yet been directly validated as a single unified mechanism in one experimental or epidemiological system. At the mechanistic level, substantial evidence indicates that heavy metals such as Pb, Cd, and As can impair intestinal barrier integrity by disrupting tight junction structure, increasing epithelial permeability, inducing oxidative stress, promoting mitochondrial dysfunction, and activating pro-inflammatory signaling pathways (11, 13, 17, 26, 27, 57). In parallel, heavy metal exposure has also been associated with gut microbiota disruption, altered microbial metabolic function, and reduced microbial buffering capacity, all of which may further aggravate epithelial injury and host vulnerability (21, 27, 37, 38).

Additional evidence suggests that heavy metals can impair host immune homeostasis through immunotoxic effects and chronic inflammatory imbalance (42, 43, 55), while metal-contaminated environments may also favor the emergence of bacterial traits linked to enhanced virulence, metal tolerance, and antibiotic resistance (52–54). These observations provide support for the biological plausibility that chronic mixed-metal exposure could create conditions conducive to opportunistic pathogen colonization and persistence. The complete sequence linking environmental metal exposure to intestinal barrier failure, dysbiosis, immune imbalance, altered pathogen behavior, and ultimately clinically increased susceptibility to opportunistic infection should be interpreted as an integrative conceptual framework rather than a definitively validated mechanistic pathway. Some components of this cascade are supported by direct experimental evidence, whereas the connections between these components, particularly in the context of chronic mixed-metal exposure in human populations, remain partly inferential. Therefore, this model is intended to synthesize current mechanistic and toxicological evidence, identify biologically coherent links, and highlight priorities for future validation in animal models, longitudinal human biomonitoring, and exposure-informed infection studies.

### Future perspectives

As discussed in this study, further research is needed to develop animal models to accurately replicate exposure scenarios encountered in humans will provide a valid model of how complex mixtures of Cd, Pb, and As effect human health in order to help inform possible future therapeutic interventions. Long-term, chronic, low-dose, oral exposure of multiple mixtures of heavy metals will be needed to simulate this type of exposure; therefore, the establishment of an animal model is needed to allow for subsequent pathogen challenges in order to determine how the complex mixture of heavy metals leads to altered susceptibility to infections, severity of disease, and host survival. In addition, a comprehensive analysis of the effects of heavy metals on intestinal tissues via spatial transcriptomics, proteomics, and metabolomics will produce valuable data to characterize how heavy metal exposure affects gene expression profiles, protein expression profiles, and metabolite profiles in intestinal epithelial, immune, and stromal cells which will help identify specific signaling pathways and molecular targets affected by heavy metals, which will aid in the development of novel therapeutic interventions.

As described above, it will also be essential to determine the effectiveness of various interventions. These may include, for example, specific combinations of probiotics or prebiotics to improve intestinal barrier function and restore a healthy microbiota, in addition to dietary supplementation with antioxidants and specific nutrients (e.g., selenium and zinc). Animal models must be used to validate whether the various interventions will be effective in ameliorating intestinal damage, restoring immune homeostasis and reducing susceptibility to pathogen challenge in an environment with chronic and unavoidable exposure to heavy metals.

## Conclusion

We have provided various pieces of evidence from environmental monitoring and ecological field studies to understand how long-term exposure to Pb and Cd and As increases the risk for intestinal infection in complex geochemical environments such as Yunnan Province. The evidence comes from a variety of fields including, but not limited to, environmental monitoring, cellular biology, microbiology, and immunology. We have provided an evidence-based discussion of how Pb and Cd and As affect the intestinal barrier and, thereby, the establishment of an intestinal dysbiosis and impaired immune function. Cumulative exposure to heavy metals from the environment limits epithelial barrier function, alters the normal intestinal microbiota, and alters immune function, resulting in an environment conducive to infection with pathogens such as *Pseudomonas aeruginosa*. A critical element of this paper is the proposition of a multi-level causal model to explain how environmental exposure to Pb and Cd and As affects host–pathogen interactions. An apparent self-sustaining cycle has been established that includes heightened oxidative stress, damage to the intestinal epithelium, disturbance of the normal intestinal microbiota, and dysregulation of the immune system. This cycle contributes to the increased risk for infection in susceptible individuals. However, despite growing evidence, several limitations remain. Current studies are largely fragmented, with insufficient longitudinal human data, limited integration across biological scales, and a lack of standardized models to evaluate combined metal toxicity. In particular, the synergistic or antagonistic effects of mixed-metal exposure and their dose–response relationships require further clarification.
